# Carbon source modulates the antifungal and antibiofilm activities of *Lactiplantibacillus plantarum* S61 supernatants against *Rhodotorula glutinis* UMP22

**DOI:** 10.1016/j.crfs.2025.101272

**Published:** 2025-12-08

**Authors:** Sara Moumnassi, Adem Gharsallaoui, Mohamed Brahmi, Nour Eddine Bentouhami, Meryem Idrissi Yahyaoui, Mohamed Taibi, Bouchra El Guerrouj, Reda Bellaouchi, Emilie Dumas, Abdeslam Asehraou

**Affiliations:** a Laboratory of Bioresources, Biotechnology, Ethnopharmacology and Health, Faculty of Sciences, Mohammed Premier University, Oujda, 60000, Morocco; b Univ Lyon, Université Claude Bernard Lyon 1, CNRS, LAGEPP UMR 5007, Villeurbanne, F-69100, France; c Oriental Center of Water Sciences and Technologies, Mohammed Premier University, Oujda, 60000, Morocco

**Keywords:** *Lactiplantibacillus plantarum* S61, Cell-free supernatant (CFS), *Rhodotorula glutinis*, Antifungal and antibiofilm activity, Natural biopreservation, Carbon source modulation

## Abstract

Natural metabolites produced by lactic acid bacteria (LAB) are emerging as sustainable alternatives to synthetic antifungal agents. This study evaluated the antifungal and antibiofilm activities of cell-free supernatants (CFSs) derived from *Lactiplantibacillus plantarum* S61, cultivated with glucose (CFS-Gl) or arabinose (CFS-Ar), against the spoilage and opportunistic yeast *Rhodotorula glutinis* UMP22. Antifungal activity was determined by agar diffusion and MIC/MFC assays, while stability was assessed under variable temperature, pH, and enzymatic conditions. Membrane permeability, evaluated through propidium iodide uptake, confirmed that CFSs disrupt fungal cell integrity, indicating a multifactorial mode of action involving organic acids and protease-sensitive peptides.

CFS-Ar exhibited significantly higher antifungal potency than CFS-Gl, achieving an inhibition zone of 36.7 ± 0.4 mm. When combined with sub-inhibitory concentrations of cycloheximide (MIC/2 = 15.5 μg/mL; MIC/3 = 10.3 μg/mL), both CFSs produced a synergistic effect, achieving near-complete growth suppression while lowering the required antifungal dose. In addition, both CFSs demonstrated strong preventive and curative antibiofilm activities across a wide range of temperatures (4–37 °C), concentrations, and exposure durations. The persistence of activity under refrigeration highlights their relevance for food preservation.

Overall, these findings identify *L. plantarum* S61 as a promising source of thermostable, pH-tolerant bioactive metabolites with dual antifungal and antibiofilm functions. The observed synergy between CFSs and conventional antifungals offers an innovative, eco-friendly, and resistance-mitigating strategy for controlling spoilage yeasts and emerging fungal contaminants in food and health-related applications.

## Highlights

• Arabinose enhances the antifungal activity of *Lactiplantibacillus plantarum* S61.
• Cell-free supernatants act through organic acids and protease-sensitive peptides.
• Combination of CFSs with cycloheximide strengthens antifungal inhibition.
• CFSs effectively prevent and eradicate *Rhodotorula glutinis* biofilms at 4–37 °C.

## Graphical abstract

**Figure:**
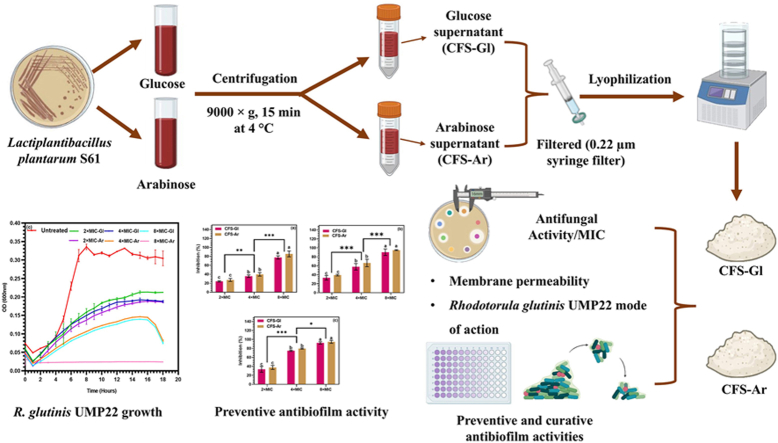

## 1 Introduction

Food spoilage remains a persistent challenge for the food industry, causing major economic losses due to product deterioration and costly recalls that undermine consumer trust (Bento de Carvalho et al., 2024). Among the microorganisms implicated, *Rhodotorula* species have recently emerged as noteworthy contaminants. Once regarded as environmental commensals, these yeasts are now recognized as opportunistic pathogens frequently isolated from food, hospital environments, and medical devices (Ioannou et al., 2019; Xiao et al., 2018). The treatment of *Rhodotorula glutinis* infections is particularly problematic because of its intrinsic resistance to commonly used antifungal agents. Several studies have reported high minimum inhibitory concentrations (MICs) for azole antifungals such as fluconazole and itraconazole, rendering them largely ineffective (Galán-Sánchez et al., 1999; Nunes et al., 2013). Moreover, resistance to triazoles and echinocandins further limits therapeutic options, leaving amphotericin B as one of the few agents with consistent *in vitro* efficacy, albeit with considerable toxicity concerns (Gharaghani et al., 2020). *Rhodotorula* is now recognized as the third most common yeast in blood cultures and is frequently recovered from the hands of hospital staff and patients (Gomez-Lopez et al., 2005), underscoring its dual relevance to both food safety and public health.

A hallmark of *Rhodotorula* pathogenicity is its strong ability to form biofilms, especially on plastic surfaces and medical devices such as central venous catheters and bronchoscopes (Hirano et al., 2022). Biofilm formation promotes persistence, enhances environmental survival, and confers protection against antifungal agents and disinfectants, complicating eradication efforts. Among *Rhodotorula* species, *R. glutinis* stands out for its robust biofilm-producing capacity, which not only contributes to food spoilage but also supports its role as an opportunistic pathogen in clinical settings. This biofilm-mediated resilience, combined with antifungal resistance, accounts for the emergence of *R. glutinis* as a problematic contaminant at the intersection of the food industry and healthcare environments (Gharaghani et al., 2020).

The use of lactic acid bacteria (LAB) with antifungal properties offers a promising natural alternative to chemical preservatives, given their Generally Recognized As Safe (GRAS) status and their long-standing application in food fermentations (Crowley et al., 2012). The antifungal potential of LAB in food preservation has been widely documented, with inhibitory effects attributed to the production of diverse metabolites such as organic acids, fatty acids, reuterin, hydrogen peroxide (H_2_O_2_), and proteinaceous compounds including bacteriocins (Siedler et al., 2019). Among these strains, *Lactiplantibacillus plantarum* S61 (originally isolated from traditionally fermented olives) has exhibited strong antifungal and antibacterial activities, particularly through its cell-free supernatant (CFS). This CFS has demonstrated significant inhibitory effects against spoilage microorganisms such as *R. glutinis* and *Listeria monocytogenes* (Abouloifa et al., 2022).

Importantly, the composition and antifungal potency of LAB supernatants can be modulated by environmental factors, notably the type of carbon source available during fermentation. Previous studies have shown that specific prebiotic substrates, such as xylo-oligosaccharides (XOS), can enhance the production of antifungal compounds in *L. plantarum* S61 compared to conventional sugars like glucose (Abouloifa et al., 2020; Yahyaoui et al., 2025). However, there remains a lack of studies directly comparing the effects of distinct monosaccharides, particularly glucose and arabinose, a non-conventional pentose, on the bioactivity of *L. plantarum* S61 supernatants.

In this context, the present study investigates how glucose and arabinose as carbon sources influence the antifungal and antibiofilm properties of lyophilized cell-free supernatants derived from *L. plantarum* S61, specifically against *Rhodotorula glutinis* UMP22. The novelty of this work lies in its dual focus. First, on evaluating arabinose as an unconventional carbon source that may reshape the antifungal and antibiofilm potential of *L. plantarum* S61, and second, on elucidating these effects against *R. glutinis*, a yeast species for which such studies remain scarce compared to the more frequently examined *R. mucilaginosa* (Gharaghani et al., 2020; Nunes et al., 2013). The influence of carbon source on *L. plantarum* S61 metabolite production and its specific interaction with *R. glutinis* biofilms provides new mechanistic insights into LAB-derived metabolites as potential natural bio-preservatives for enhancing food safety and extending shelf life.

## 2 Materials and methods

### 2.1 Materials

De Man, Rogosa, and Sharpe (MRS) medium, yeast extract, and bacteriological agar (type E) were obtained from Biokar Diagnostics (Beauvais, France). Individual MRS constituents (peptone, meat extract, polysorbate 80 (Tween 80), ammonium citrate, sodium acetate, magnesium sulfate, manganese sulfate, and dipotassium phosphate) as well as glucose and arabinose were purchased from Sigma-Aldrich (Merck KGaA, Darmstadt, Germany). Cycloheximide, trypsin, pepsin, catalase, proteinase K, crystal violet, and propidium iodide were also sourced from Sigma-Aldrich. Unless otherwise specified, all reagents were of analytical grade. Solutions were prepared in ultrapure water, sterilized by 0.22 μm filtration, and used immediately or stored at 4 °C.

### 2.2 Strains and growth conditions

*Lactiplantibacillus plantarum* S61, previously identified at the Laboratory of Bioresources, Biotechnologies, Ethnopharmacology, and Health (LBBEH, Oujda, Morocco) (Abouloifa et al., 2020), was originally isolated from traditionally fermented olives and selected for its enzymatic and probiotic properties. The strain was cultured in MRS broth and incubated at 37 °C for 24 h. The fungal strain *Rhodotorula glutinis* UMP22 (ON209167.1) was grown in yeast extract–glucose (YEG) broth containing 5 g/L yeast extract and 10 g/L glucose. Prior to use, *R. glutinis* UMP22 suspensions were adjusted to an optical density (OD_600_) corresponding to a 0.5 McFarland standard.

### 2.3 Production of cell-free supernatant (CFS) from *L. plantarum* S61

*L. plantarum* S61 was inoculated into modified MRS media containing either glucose or arabinose as the sole carbon source, each at a concentration equivalent to that in commercial MRS (20 g/L), with an initial OD_600_ = 0.06. After incubation at 37 °C for 24 h, cultures were centrifuged at 9000×*g* for 15 min at 4 °C (Ohaus, France). The resulting supernatant was filtered through a 0.22 μm membrane and evaluated for inhibitory activity. The glucose- and arabinose-derived supernatants (designated CFS-Gl and CFS-Ar, respectively) were frozen at −20 ± 2 °C for 24 h and then lyophilized.

### 2.4 Antifungal activity

The antifungal activity of CFS-Gl and CFS-Ar (10 % w/v) was assessed against *R. glutinis* UMP22. Yeast cultures were maintained on YEG agar, and cell suspensions were standardized to 10^6^ cells/mL based on OD_600_ measurements using a UV–visible spectrophotometer.

### 2.5 Well diffusion assay

The antifungal activity of CFS-Gl and CFS-Ar was determined by the agar well diffusion method. Assays were performed in sterile 90 mm Petri dishes. For each test, 80 μL of sample was introduced into wells punched into the agar. MRS medium served as the negative control, while cycloheximide (0.01 mg/mL; Sigma-Aldrich, USA) was used as the positive control. After incubation, the diameter of the inhibition zones was measured in millimeters using a precision digital caliper. All experiments were performed in triplicate.

### 2.6 Determination of the minimum inhibitory concentration (MIC)

The MIC values of CFS-Gl and CFS-Ar were determined by the microdilution method in 96-well microplates. YEG medium supplemented with 0.15 % agar was used. Serial dilutions of the samples (50 mg/mL to 0.39 mg/mL) were prepared, and 50 μL of standardized inoculum (10^6^ cells/mL) was added to each well. Microplates were incubated at 25 °C for 24 h. To detect growth, 20 μL of resazurin solution (0.015 % w/v) was added to each well, followed by a 2 h incubation at 25 °C. A color change from blue to pink indicated metabolic activity. The MIC was defined as the lowest concentration showing no color change. All assays were performed in duplicate.

### 2.7 Determination of the minimum fungicidal concentration (MFC)

To determine the MFC, 3 μL from each well that remained blue (indicating no metabolic activity) was aseptically transferred to YEG agar plates and incubated at 25 °C for 24 h. The MFC was defined as the lowest concentration at which no visible fungal growth was observed.

### 2.8 Effect of temperature, pH, and proteolytic enzymes on the antifungal activity of CFS

The effect of pH on the anti-*R. glutinis* UMP22 activity of CFS-Gl and CFS-Ar (10 % w/v) was evaluated by adjusting the pH to 4, 5, or 7 using 1 M NaOH. Residual antifungal activity was assessed by measuring inhibition zones. For thermal stability, the method of Yang et al. (2012) was applied with minor modifications. CFS-Gl and CFS-Ar were heated in a water bath at 80 °C for 10 or 30 min, and at 100 °C for 30 min or 3 h. The residual activity was compared to that of untreated controls. Enzymatic sensitivity was tested by treating the CFS samples with pepsin, trypsin, catalase, or proteinase K (5.0 mg/mL each). After incubation at 37 °C for 3 h, enzymes were inactivated by heating the samples at 100 °C for 5 min. The remaining antifungal activity was evaluated by measuring inhibition zones with a digital caliper. Untreated CFS served as the control.

### 2.9 pH-dependent growth response of *R. glutinis* UMP22 in the presence and absence of CFS

As the CFS from *L. plantarum* S61 exhibited an acidic pH (∼4.2), the growth and survival of *R. glutinis* UMP22 were studied across a range of pH values to determine whether inhibition resulted solely from acidity or also from antifungal metabolites. YEG medium was adjusted to pH 4.0, 5.0, 7.0, and 8.0 using 1 M HCl or 1 M NaOH. Growth kinetics were monitored in 96-well microplates, and pH values of control and CFS-treated media were recorded before inoculation. After 18 h of incubation at 25 °C, final measurements of pH and growth were performed. The viability of untreated and CFS-treated samples was also evaluated.

### 2.10 Propidium iodide (PI) uptake assay

The PI uptake assay was performed following Han et al. (2022) with minor modifications to quantify membrane permeability changes in *R. glutinis* UMP22 upon exposure to *L. plantarum* S61 supernatants (CFS-Gl and CFS-Ar). *R. glutinis* UMP22 was cultured overnight in YEG broth at 25 °C, harvested by centrifugation at 10,000×*g* for 10 min at 4 °C, washed twice, and resuspended in phosphate-buffered saline (PBS, pH 7.2) to 10^6^ CFU/mL (OD_600_ standardized).

Aliquots (100 μL) of yeast suspension were transferred to wells of a sterile black 96-well microplate. Equal volumes (100 μL) of CFS-Gl or CFS-Ar were added to reach final concentrations of 2 × , 4 × , and 8 × MIC. After incubation for 3 h at 25 °C, PI was added to a final concentration of 20 μM, followed by 10 min incubation in the dark at room temperature.

Optical density was measured at 600 nm to normalize for cell density, and fluorescence intensity was recorded (excitation = 535 nm, emission = 617 nm) using a Varioskan Lux microplate reader (Thermo Scientific, France). Membrane permeability was expressed as relative fluorescence units (RFU) normalized to OD_600_. Results were compared across concentrations, with untreated and heat-treated samples serving as controls for intact and fully permeabilized membranes, respectively.

The PI uptake was normalized using the formula:

(1) FF0=FluorescenceaftertreatmentOD600FluorescenceofuntreatedsampleOD600

### 2.11 Assessment of *L. plantarum* S61 CFS combined with Sub-MIC concentrations of cycloheximide against *R. glutinis* UMP22

This experiment aimed to explore whether the combination of *L. plantarum* S61 cell-free supernatants with sub-inhibitory concentrations of cycloheximide could enhance antifungal activity against *R. glutinis* UMP22, as a preliminary step toward reducing reliance on high doses of synthetic and expensive antifungal agents. The combined antifungal effect of CFSs and a commercial cycloheximide was evaluated against *R. glutinis* UMP22. Yeast suspensions were adjusted to 10^6^ CFU/mL in YEG medium and distributed into 96-well microplates. CFS-Gl and CFS-Ar were tested at concentrations corresponding to 2 × , 4 × , and 8 × the MIC. Cycloheximide was applied at sub-inhibitory levels equivalent to MIC/2 (C1: 15.5 μg/mL) and MIC/3 (C2: 10.3 μg/mL).

For each CFS concentration tested, the following conditions were evaluated: CFS alone, cycloheximide alone (C1 or C2), and combinations of CFS with cycloheximide (C1 or C2). Untreated wells and medium-only wells served as controls. Microplates were incubated at 25 °C for 18 h under identical conditions, and fungal growth was kinetically monitored by measuring the optical density at 600 nm using a microplate reader.

### 2.12 Biofilm formation under different temperatures

The biofilm-forming ability of *R. glutinis* UMP22 was assessed using a modified method of Abou Elez et al. (2023). Biofilm development was evaluated in sterile 96-well polystyrene microplates at three storage temperatures: 4 °C, 25 °C, and 37 °C. Yeast suspensions were prepared in YEG broth, adjusted to a 0.5 McFarland standard (∼10^6^ CFU/mL), and 100 μL aliquots were transferred to each well. Each temperature condition was tested in triplicate. Plates were incubated for 24 h at 4 °C (plate I), 25 °C (plate II), and 37 °C (plate III).

Three wells in each plate containing YEG broth only served as negative controls. After incubation, wells were gently washed twice with 200 μL of phosphate-buffered saline (PBS, pH 7.2) to remove non-adherent cells. Excess liquid was removed by inversion and blotting with sterile absorbent paper. Biofilms were fixed by adding 150 μL of ethanol to each well and incubating for 20 min. Subsequently, wells were stained with 150 μL of 0.1 % (w/v) crystal violet for 15 min at room temperature. After staining, wells were rinsed twice with PBS and air-dried for 1 h.

To quantify biofilm biomass, 150 μL of 95 % ethanol was added to each well and incubated for 45 min to solubilize the retained dye. Absorbance was measured at 570 nm using a microplate reader.

Biofilm formation by *R. glutinis* UMP22 was classified according to the following criteria.

• **Negative**: OD_570_ ≤ OD_nC_
• **Weak**: OD_nC_ < OD_570_ ≤ 2 × OD_nC_
• **Moderate**: 2 × OD_nC_ < OD_570_ ≤ 4 × OD_nC_
• **Strong**: OD_570_ > 4 × OD_nC_

where OD_570_ corresponds to the optical density of the biofilm at 570 nm, and OD_nC_ represents the optical density of the negative control (YEG medium) at the same wavelength.

### 2.13 Antibiofilm activity

#### 2.13.1 Preventive antibiofilm assay

A fungal suspension of *R. glutinis* UMP22 adjusted to 0.5 McFarland (10^6^ CFU/mL) was co-incubated with CFS-Gl and CFS-Ar at concentrations corresponding to 2 × , 4 × , and 8 × MIC. A volume of 100 μL of the mixture was dispensed into microtiter plates as described previously. Plates were incubated at 4 °C, 25 °C, and 37 °C for 24 h to assess the preventive antibiofilm activity of the CFS samples at different temperatures.

#### 2.13.2 Curative antibiofilm assay

To evaluate the curative effect of CFS-Gl and CFS-Ar on pre-formed *R. glutinis* UMP22 biofilms, a fungal suspension (10^6^ CFU/mL) was inoculated into 96-well microplates following the same procedure as described above. After 24 h of incubation, wells were gently washed twice with 200 μL of PBS (pH 7.2) to remove non-adherent cells and blotted to eliminate residual liquid. CFS-Gl and CFS-Ar were then added at concentrations of 2 × , 4 × , and 8 × MIC. The inhibitory effect on mature biofilms was evaluated after 2, 4, and 24 h of incubation.

#### 2.13.3 Quantification of biofilm inhibition

Biofilm inhibition under both preventive and curative conditions was quantified by measuring the optical density at 570 nm using a microplate reader (Multiskan Sky, Thermo Scientific). Results were expressed as relative reductions in biofilm biomass compared to the untreated control.

The percentage of biofilm inhibition was calculated using the following formula:

(2) $$\text{Biofilm}\;\text{inhibition}\;\text{rate}\;\left(\%\right)=\frac{OD\left(R.\;glutinis\right)-OD\left(CFS\right)}{OD\left(R.\;glutinis\right)-OD\left(control\right)}\times 100$$

Where OD_(*R.glutinis*)_ referred to the optical density of the biofilm formed by *R. glutinis* UMP22, OD_(CFS)_ is the optical density measured in the presence of CFS-Gl or CFS-Ar, and OD_(control)_ was the optical density of the YEG medium (blank control).

### 2.14 Statistical analysis

All experiments were performed in triplicate, and the results are expressed as mean ± standard deviation (SD). Statistical differences among groups were evaluated using one-way analysis of variance (ANOVA), followed by Tukey's honestly significant difference (HSD) post hoc test for multiple comparisons. Differences were considered statistically significant at *p* < 0.05. All analyses were conducted using OriginPro 2018 (OriginLab Corporation, Northampton, MA, USA).

## 3 Results and discussion

### 3.1 Examination of the antifungal activity of CFS against *R. glutinis*

To evaluate the antifungal potential of the cell-free supernatants (CFS) produced by *L. plantarum* S61, inhibition zone, MIC, and MFC assays were performed against *R. glutinis* UMP22. The results are summarized in Table 1. Examination of the inhibition zones revealed that both CFS obtained from glucose (CFS-Gl) and arabinose (CFS-Ar) displayed significantly higher antifungal activity than the reference antifungal cycloheximide, demonstrating the strong inhibitory capacity of metabolites secreted by *L. plantarum* S61. Furthermore, a marked difference was observed between the two supernatants, emphasizing the influence of the fermentation substrate on antifungal efficacy.

**Table 1:** Antifungal activity of CFS-Gl and CFS-Ar against *R. glutinis* UMP22 expressed as inhibition zone (mm), MIC (mg/mL), and MFC (mg/mL). Data are presented as mean ± SD (n = 3). Letters (a-b) refer to statistically significant differences between the groups (*p* < 0.05).

	Antifungal activity (mm)	MIC (mg/mL)	MFC (mg/mL)
CFS-Gl	32.07 ± 0.6^b^	2.34 ± 1.1^b^	12.50 ± 0.0^b^
CFS-Ar	36.71 ± 0.43^a^	1.17 ± 0.55^a^	9.40 ± 2.1^a^
Cycloheximide	26.87 ± 0.82	0.03 ± 0.0	0.06 ± 0.0

CFS-Gl exhibited a clear inhibitory effect against *R. glutinis* UMP22, with an inhibition zone of 32.07 ± 0.60 mm, in line with previous findings reporting the antifungal potential of *L. plantarum* S61 supernatants enriched in organic acids, peptides, and other bioactive molecules (Abouloifa et al., 2022). When arabinose was used as the carbon source, the antifungal activity was significantly enhanced (*p* < 0.05), with CFS-Ar producing a larger inhibition zone of 36.71 ± 0.43 mm. This improvement can be attributed to the metabolic shift from glucose (metabolized mainly through the Embden–Meyerhof pathway) to arabinose (a pentose primarily processed via the phosphoketolase pathway), which has been shown to increase the production of lactic and acetic acids (Gobbetti et al., 2000). The presence of acetic acid is known to markedly strengthen the antifungal effect of LAB-derived supernatants (Guimarães et al., 2018), while a synergistic interaction between lactic and acetic acids has also been suggested to enhance overall antifungal efficacy (Cabo et al., 2002).

In our previous work, quantitative HPLC analyses conducted on glucose-based *L. plantarum* S61 supernatants revealed the presence of lactic acid as the major component (121.00 ± 0.80 mM), accompanied by acetic, citric, and oxalic acids at concentrations of 11.12 ± 0.14, 7.14 ± 0.12, and 4.24 ± 0.21 mM, respectively. When glucose was replaced with xylo-oligosaccharides (XOS), the CFS of *L. plantarum S61* exhibited stronger inhibitory effects against *R. glutinis*, which were attributed to the appearance of new organic acids such as malic acid, as well as a significant increase in the concentrations of acetic, citric, and oxalic acids (Yahyaoui et al., 2025). These findings collectively highlight the crucial role of the carbon source in modulating the metabolic profile and antifungal potency of *L. plantarum* S61.

Quantitative data from MIC and MFC determinations (Table 1) corroborate these observations. Both parameters were significantly influenced by the carbon source (*p* < 0.05), consistent with the inhibition zone results. Specifically, a 50 % decrease in MIC and a 25 % reduction in MFC were recorded when *L. plantarum* S61 was cultured with arabinose instead of glucose. These results provide quantitative evidence that optimizing the fermentation substrate is a key factor in enhancing the antifungal performance of *L. plantarum* S61 CFS against *R. glutinis* UMP22.

### 3.2 Effect of heat, pH, and proteolytic enzymes on the antifungal activity of CFS

Considering the potential industrial application of this approach, it was essential to examine the effects of temperature, pH, and enzymatic treatments on the antifungal activity of *L. plantarum* S61 cell-free supernatants, namely CFS-Gl and CFS-Ar, against *R. glutinis* UMP22. As shown in Table 2, both supernatants retained strong antifungal activity after heating at 80–100 °C, and no significant loss (p > 0.05) was detected even after the longest treatment (100 °C, 3 h). The slight, non-significant decrease observed under these conditions may simply reflect minor changes in the concentration of bioactive components caused by prolonged heating.

**Table 2:** Effect of temperature, pH, and enzymes on the antifungal activity (inhibition zone) of CFS-Gl and CFS-Ar. Data are presented as mean ± SD (n = **3**). Letters (a-**f**) refer to statistically significant differences between the groups (*p* < 0.05).

	Temperature effect				pH effect			Enzyme effect
	80 °C		100 °C		4	5	7	
	10 min	30 min	30 min	3 h				
**Inhibition zone (mm)**								
**CFS-Gl**	31.96 ± 0.01^a^	32.04 ± 0.13^a^	32.35 ± 0.24^a^	33.75 ± 1.09^a^	32.55 ± 0.85^a^	25.42 ± 0.77^b^	11.66 ± 0.76^c^	ND
**CFS-Ar**	36.42 ± 0.62^d^	36.48 ± 0.88^d^	36.63 ± 0.63^d^	38.41 ± 0.77^d^	36.65 ± 1.01^d^	28.88 ± 1.01^e^	14.53 ± 1.07^f^	ND

*ND: No detectable antifungal activity*.

The persistence of antifungal activity at high temperatures demonstrates that the active metabolites in CFS-Gl and CFS-Ar are highly thermostable, a key advantage for potential use in food processing operations involving pasteurization or thermal treatment. Organic acids such as lactic, acetic, and phenyllactic acids, commonly produced by *L. plantarum* strains, are known to maintain their antifungal functionality after exposure to heat (Li et al., 2023). Similarly, phenolic and other secondary metabolites from *L. plantarum* have been reported to resist thermal degradation, thus preserving their inhibitory potential under industrial processing conditions (Wang et al., 2024).

Regarding pH stability, antifungal activity was maximal at acidic pH (4–5) and decreased markedly at neutral pH (7), where inhibition zones dropped to 11.6 mm for CFS-Gl and 14.5 mm for CFS-Ar (Table 2). This pH-dependent pattern is consistent with the known mechanism of weak organic acids, which exhibit optimal antifungal effects in acidic environments. At low pH, undissociated acid molecules (such as lactic, acetic, and phenyllactic acids) can freely diffuse through fungal cell membranes. Once inside the cytoplasm, they dissociate and release protons, lowering the intracellular pH and disrupting enzymatic function, ultimately leading to growth inhibition (Belguesmia et al., 2014; Cabo et al., 2002; De Muynck et al., 2004).

Importantly, CFS-Ar consistently exhibited larger inhibition zones than CFS-Gl under all tested conditions, confirming that arabinose as a carbon source might not only increases the amount of antifungal organic acids produced by *L. plantarum* S61 but also leads to a more robust metabolite profile that remains active under stressing thermal and pH conditions.

In light of the pH-dependent behavior of the supernatants, this experiment aimed to determine whether components other than organic acids contribute to the antifungal activity of *L. plantarum* S61 CFS. To minimize the influence of organic acids, the CFS samples were first neutralized before being subjected to enzymatic treatment with pepsin, trypsin, catalase, and proteinase K. These enzymes are capable of degrading peptides or bacteriocin-like substances that may be present in the CFS.

As shown in Table 2, the application of proteolytic enzymes completely abolished the antifungal activity in both CFS-Gl and CFS-Ar. This total loss of activity following enzymatic digestion strongly suggests that peptides or bacteriocin-like compounds are primarily responsible for the residual antifungal effects observed at neutral pH. These proteinaceous components may also act synergistically with organic acids to enhance overall antifungal efficacy.

Previous work by Abouloifa et al. (2022) identified low-molecular-weight peptides (2–6 kDa) in *L. plantarum* S61 supernatants exhibiting antifungal activity against yeasts. Similar proteinaceous antifungal substances have been reported in other *L. plantarum* strains, such as ALAC-4, where protease treatment markedly reduced antifungal activity, confirming the peptide nature of the active molecules (Chen et al., 2018).

### 3.3 *R. glutinis* growth as a function of pH and culture medium composition

To better understand the antifungal mechanism of *L. plantarum* S61 cell-free supernatants, the effect of the initial pH and the composition of the culture medium on the growth kinetics of *R. glutinis* UMP22 was evaluated. Yeast cultures were grown either without treatment or in the presence of increasing concentrations of CFS-Gl or CFS-Ar. This experiment provided insights into how environmental pH and CFS composition modulate yeast growth dynamics.

**Fig. 1:**
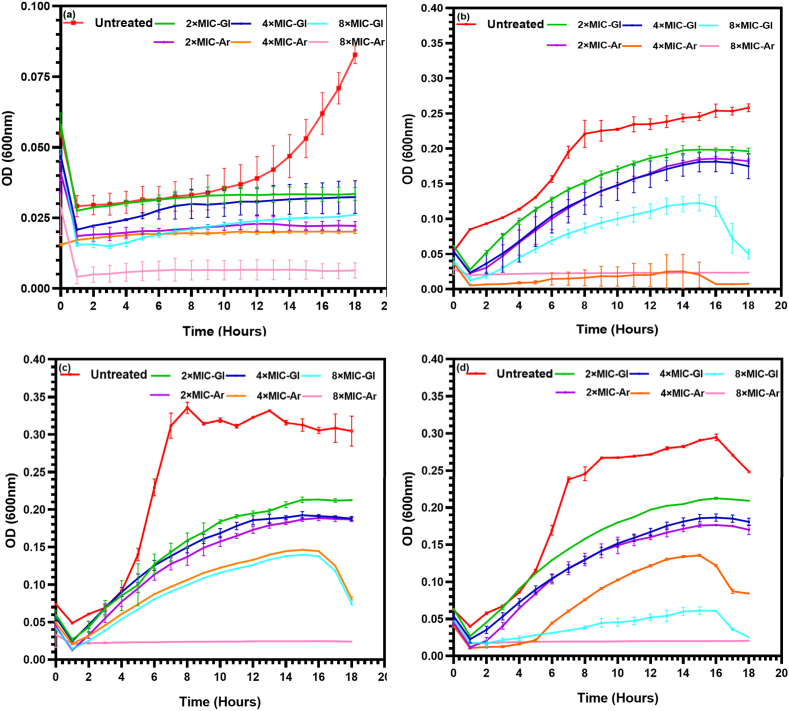
Effect of medium pH on *R. glutinis* UMP22 growth in the presence and absence of *L. plantarum* S61 cell-free supernatants (CFSs). (a) pH 4.0, (b) pH 5.0, (c) pH 7.0, (d) pH 8.0. Untreated: *R. glutinis* growth without CFS; 2 × MIC-Gl, 4 × MIC-Gl, 8 × MIC-Gl: cultures treated with 2, 4, or 8 × MIC of glucose-derived CFS; 2 × MIC-Ar, 4 × MIC-Ar, 8 × MIC-Ar: cultures treated with 2, 4, or 8 × MIC of arabinose-derived CFS. Data are presented as mean ± SD (n = 3).

As shown in Fig. 1, the growth of untreated *R. glutinis* UMP22 was strongly influenced by the initial pH. At pH 4, growth remained minimal, whereas at pH 5–8, typical sigmoidal curves developed, confirming that acidic environments are less favorable for *Rhodotorula* proliferation (Kot et al., 2017). In cultures treated with CFSs, yeast growth was markedly reduced at all pH values, with inhibition depending on both CFS concentration and the carbon source used during its production. At pH 4, the already limited growth of the control was further curtailed in the presence of CFS-Gl and CFS-Ar, indicating that, beyond acidity, antifungal metabolites present in the supernatants (principally organic acids) play a decisive role in inhibition across pH conditions.

At higher pH levels (5–8), CFS-Ar consistently outperformed CFS-Gl, with 8 × MIC almost completely suppressing growth, whereas residual proliferation was still detectable at lower concentrations.

These findings support the hypothesis that *R. glutinis* inhibition by *L. plantarum* S61 supernatants is primarily mediated by organic acids, which lower the environmental pH and disrupt intracellular metabolism, while peptides and phenolic derivatives contribute complementary fungistatic and fungicidal effects. Similar pH-dependent antifungal behaviors have been reported in other *L. plantarum* strains. For instance, *L. plantarum* UM55 supernatants displayed pH-dependent inhibitory activity against *Aspergillus* spp., with lactic, phenyllactic, hydroxyphenyllactic, and indole lactic acids identified as the main inhibitory metabolites (Guimarães et al., 2018a, Guimarães et al., 2018b).

The ability of CFS-Gl and CFS-Ar to suppress yeast growth even under varying pH conditions underscores their potential application as natural biopreservatives in food storage and processing systems.

### 3.4 Propidium iodide uptake assay

**Fig. 2:**
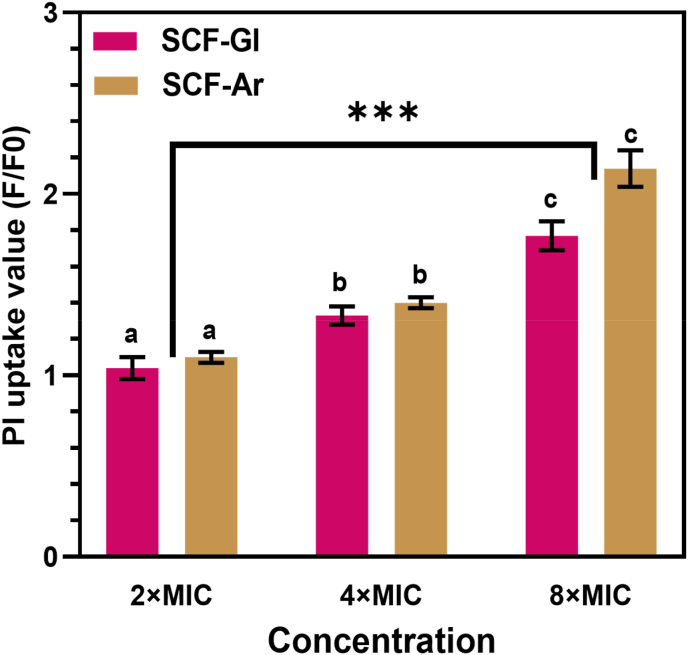
Membrane permeability of *R. glutinis* UMP22 evaluated by propidium iodide (PI) uptake after treatment with CFS-Gl and CFS-Ar. Data are presented as mean ± SD (n = 3). Different letters (a–c) and the asterisk (∗) indicate statistically significant differences between groups at p < 0.05.

To elucidate the mechanism underlying the observed antifungal activity, a propidium iodide (PI) uptake assay was performed to assess the contribution of membrane damage, as PI fluorescence reflects the loss of membrane integrity in *R. glutinis* cells. The normalized PI uptake results are presented in Fig. 2. The data revealed that exposure to CFSs induced significant membrane disruption in a dose-dependent manner. At the lowest concentration tested (2 × MIC), modest PI uptake was observed: 1.04-fold for CFS-Gl and 1.10-fold for CFS-Ar. Increasing the concentration to 4 × MIC and 8 × MIC led to a progressive rise in fluorescence intensity, indicating enhanced membrane permeability. Statistical analysis confirmed that CFS concentration had a significant effect on PI uptake (*p* < 0.05), supporting a clear concentration-dependent response.

The membrane-disruptive activity of lactic acid bacteria supernatants is a well-recognized antifungal mechanism (Liu et al., 2023; Xu et al., 2024). Such effects are associated with alterations in fungal morphology, loss of cell integrity, and increased membrane permeability. Similarly, *L. plantarum* CFSs have been shown to exert fungicidal activity against *Candida* species, where membrane disruption was identified as one of the major causes of cell death (Sharma and Srivastava, 2014).

Interestingly, although CFS-Ar exhibited greater overall antifungal activity than CFS-Gl, its effect on membrane integrity was not significantly higher (*p* > 0.05). This suggests that, while increased membrane permeability is one mechanism of action, other factors likely contribute synergistically to the antifungal effect. One such factor involves the diffusion of undissociated organic acids through the fungal membrane, leading to intracellular acidification and the collapse of the proton motive force (Schillinger and Villarreal, 2010). Moreover, the persistence of antifungal activity at pH values above the *pK*_*a*_ of these acids indicates that additional metabolites, such as peptides or phenolic derivatives, may also play a role (Sangmanee and Hongpattarakere, 2014).

### 3.5 Inhibition of *R. glutinis* UMP22 using *L. plantarum* CFSs in combination with sub-MIC cycloheximide

Given the growing interest in natural, cost-effective antifungal agents that reduce dependence on synthetic compounds, it was relevant to explore the combined use of *L. plantarum* S61 cell-free supernatants (CFSs) with low concentrations of the commercial antifungal cycloheximide. Such an approach aligns with sustainable and eco-friendly strategies for food preservation, as LAB-derived byproducts are inexpensive, safe, and widely available.

**Fig. 3:**
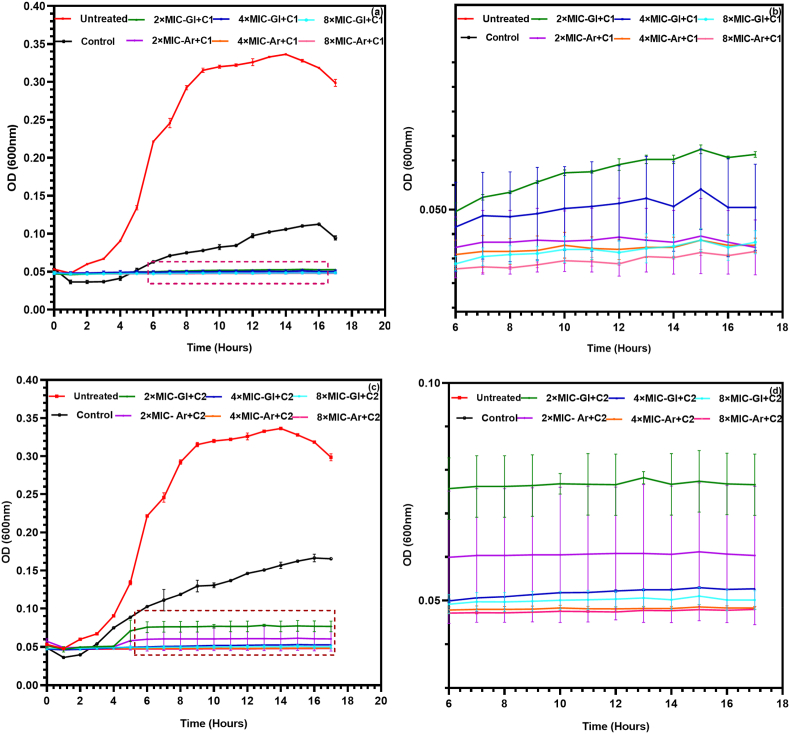
Combined effect of *L. plantarum* S61 cell-free supernatants (CFS-Gl and CFS-Ar) with sub-MIC concentrations of cycloheximide on *R. glutinis* UMP22 growth. (a) Cycloheximide at MIC/2 (15 μg/mL) combined with CFS at 2 × , 4 × , or 8 × MIC; (b) magnified view of the boxed region in (a); (c) cycloheximide at MIC/3 (10.3 μg/mL) combined with CFS at 2 × , 4 × , or 8 × MIC; (d) magnified view of the boxed region in (c). (Legend: Untreated = no treatment; Control = cycloheximide alone [C1 or C2]; 2 × /4 × /8 × MIC-(Gl or Ar) + C1/C2 = CFS combined with cycloheximide at the indicated level). Data are presented as mean ± SD (n = 3).

**Fig. 4:**
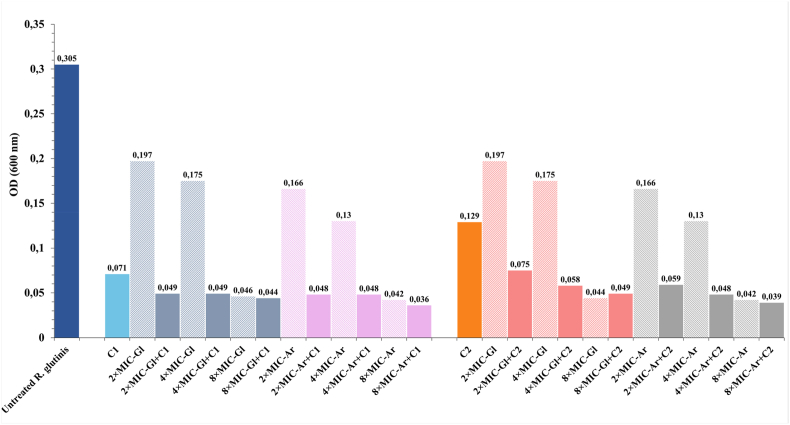
Final growth (OD_600_) of *R. glutinis* UMP22 at the stationary phase following treatment with *L. plantarum* S61 cell-free supernatants (CFS-Gl and CFS-Ar), alone or in combination with sub-MIC concentrations of cycloheximide. (Untreated = no treatment; 2 × /4 × /8 × MIC-(Gl or Ar) = CFS alone; 2 × /4 × /8 × MIC-(Gl or Ar) + C1/C2 = CFS combined with cycloheximide at C1 [MIC/2, 15 μg/mL] or C2 [MIC/3, 10.3 μg/mL]).

In this experiment, sub-MIC concentrations of cycloheximide, MIC/2 (C1) and MIC/3 (C2), were combined with CFS-Gl and CFS-Ar at concentrations of 2 × MIC, 4 × MIC, and 8 × MIC. The growth kinetics of *R. glutinis* UMP22 under these different conditions are presented in Fig. 3, while overall inhibition patterns are summarized in Fig. 4.

The untreated yeast cultures (red lines) exhibited typical exponential growth, reaching an optical density (OD_600_) of approximately 0.35 after 12 h. Treatment with cycloheximide alone resulted in partial inhibition, with growth delay at C1 (MIC/2) and a weaker effect at C2 (MIC/3), confirming that sub-inhibitory concentrations are insufficient to fully prevent proliferation. Although these sub-MIC levels of cycloheximide caused stronger inhibition than 2 × MIC and 4 × MIC of either CFS applied alone, the inhibitory effect of CFSs increased markedly at 8 × MIC, surpassing that of cycloheximide alone.

Remarkably, the combined application of CFSs with sub-MIC cycloheximide produced a substantial synergistic effect. Even at 2 × MIC of CFS, the combinations led to a pronounced reduction in OD_600_ compared with individual treatments, while 4 × MIC and 8 × MIC combinations almost completely abolished yeast growth (Fig. 3, Fig. 4). Statistical analysis confirmed that these effects were significant (*p* < 0.05) compared to untreated and cycloheximide-only controls. The enhancement of antifungal activity was more pronounced at MIC/2 than at MIC/3, yet both combinations performed better than either agent alone. A significant difference (*p* < 0.05) was also observed between CFS-Gl and CFS-Ar, the latter consistently showing superior inhibitory activity, consistent with earlier observations that arabinose enhances the production of antifungal metabolites by *L. plantarum* S61.

This enhanced antifungal activity likely resulted from complementary and potentially synergistic mechanisms between the two agents. Cycloheximide, on the one hand, was widely reported to act intracellularly by blocking ribosomal translocation, thereby inhibiting protein synthesis and compromising the yeast's ability to activate stress response pathways (Schneider-Poetsch et al., 2010). In parallel, given that among the mechanisms of action of CFS-Ar and CFS-Gl on *R. glutinis*, membrane disruption observed through PI uptake assays represented a key mode of action, it can be assumed that the synergistic effect could have been supported by CFSs-induced alterations in membrane permeability, which might have facilitated the intracellular penetration of cycloheximide and amplified its inhibitory effect. Crowley et al. (2012) reported that lactic acid and other organic acids enhanced azole activity against *Candida albicans*, enabling reduced drug dosages without compromising efficacy. Likewise, antimicrobial peptides combined with fungicides were shown to suppress resistance and increase overall fungicidal potency (Siedler et al., 2019).

To the best of our knowledge, this is the first study to demonstrate a potential synergistic inhibition of *R. glutinis* resulting from the combined effects of translational arrest (via cycloheximide) and membrane destabilization (via *L. plantarum* metabolites). This dual mechanism likely exceeds the adaptive capacity of *R. glutinis*, leading to rapid and sustained inhibition of fungal growth. However, the proposed synergistic interaction necessitates further investigation to provide a deeper understanding of the underlying mechanisms.

### 3.6 *R. glutinis* biofilm formation

Understanding the impact of temperature on biofilm formation by *R. glutinis* is essential, given its relevance to both clinical and food industry settings. These environments encompass refrigeration conditions (4 °C), ambient temperature (25 °C), and physiological temperature (37 °C). Evaluating biofilm behavior under these conditions provides critical insights into the ecological adaptability, spoilage potential, and pathogenic characteristics of this yeast.

**Fig. 5:**
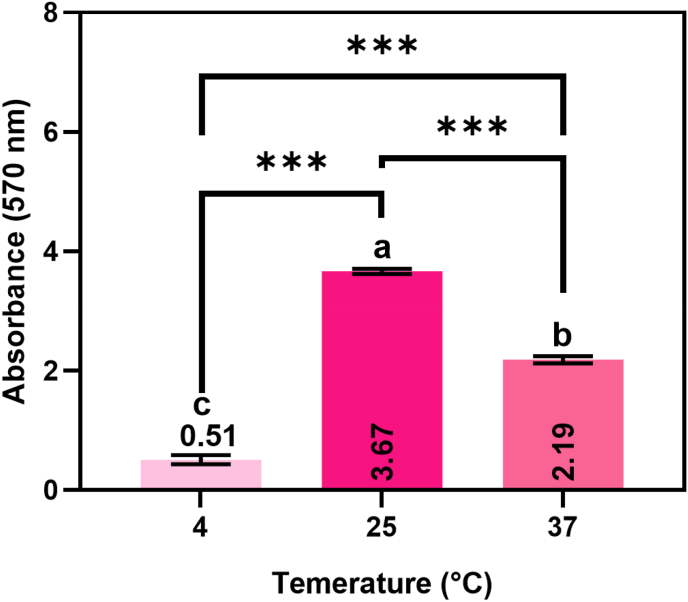
*Rhodotorula glutinis* UMP22 biofilm formation at different temperatures (4 °C, 25 °C, and 37 °C). Data are presented as mean ± SD (n = 4)**.** Different lowercase letters (a–c) and the asterisk (∗) indicate statistically significant differences between groups at p < 0.05.

The formation of *R. glutinis* UMP22 biofilms at 4 °C, 25 °C, and 37 °C (Fig. 5) clearly demonstrates that temperature exerts a significant influence on both adhesion and biofilm maturation. At 25 °C, *R. glutinis* exhibited the highest biofilm-forming activity (OD_570_ = 3.67), indicating that these conditions are optimal for extracellular matrix (ECM) production and stable surface colonization. This finding suggests that 25 °C represents a metabolic optimum in which biosynthetic pathways, particularly those involved in exopolysaccharide synthesis, are maximally activated to promote biofilm development.

At 37 °C, biofilm formation decreased (OD_570_ = 2.19) but remained within the “strong” classification range. From a clinical standpoint, this observation is noteworthy: the ability of *R. glutinis* to form substantial biofilms at body temperature implies its potential to colonize host-associated environments, contributing to its opportunistic behavior. Biofilms formed under such conditions may persist on medical devices or mucosal surfaces, thereby increasing the risk of invasive infections in immunocompromised individuals (Miglietta et al., 2015). Notably, *R. glutinis* was also capable of developing a moderate biofilm at 4 °C (OD_570_ = 0.51), underscoring its resilience under cold stress conditions. This ability to persist in refrigerated environments aligns with its established role as a spoilage organism in chilled food products. Previous studies have shown that *R. glutinis* can withstand extended refrigeration by maintaining membrane integrity and activating stress adaptation mechanisms, allowing the cells to remain viable and metabolically active in cold food environments (Gabier et al., 2005; Zhang et al., 2007).

### 3.7 Antibiofilm activity

To comprehensively and comparatively evaluate the antibiofilm potential of CFS-Gl and CFS-Ar, two complementary experimental approaches were applied. The first aimed to assess the preventive effect, corresponding to the inhibition of biofilm formation during the early stages of growth, while the second focused on the curative effect, referring to the disruption of preformed mature biofilms. These two distinct assays provided a more complete understanding of the capacity of *L. plantarum* S61 metabolites to interfere with *R. glutinis* biofilm development at different stages of maturation.

#### 3.7.1 Preventive antibiofilm effect

**Fig. 6:**
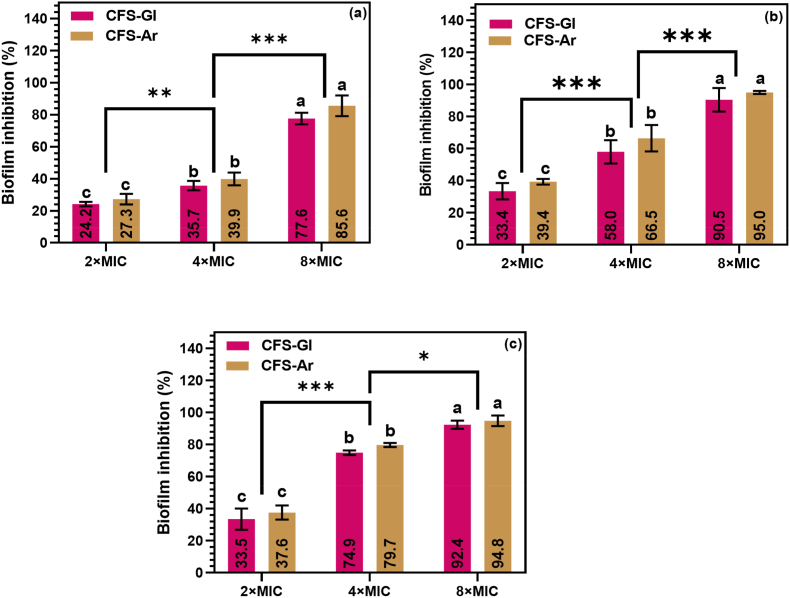
Preventive effect of *Lactiplantibacillus plantarum* S61 cell-free supernatants (CFS-Gl and CFS-Ar) on *Rhodotorula glutinis* UMP22 biofilm formation. (a) incubation at 4 °C; (b) incubation at 25 °C; (c) incubation at 37 °C. Data are presented as mean ± SD (n = 4)**.** Different lowercase letters (a–c) and the asterisk (∗) indicate statistically significant differences between groups at *p* < 0.05.

The antibiofilm activity of *L. plantarum* S61 cell-free supernatants (CFS-Gl and CFS-Ar) was evaluated to determine their potential to prevent the formation of *R. glutinis* biofilms. The influence of the carbon source on the inhibitory performance was also investigated. Given the ability of *R. glutinis* to form biofilms across a wide temperature range, the preventive effects of CFS-Gl and CFS-Ar were assessed at three concentrations (2 × MIC, 4 × MIC, and 8 × MIC) and three incubation temperatures (4, 25, and 37 °C), as shown in Fig. 6.

Statistical analysis revealed a clear dose-dependent inhibition of biofilm formation for both CFSs at all tested temperatures (Fig. 6a–c). At 2 × MIC, inhibition remained moderate, while increasing the concentration to 4 × MIC significantly (p < 0.05) enhanced biofilm suppression, and 8 × MIC resulted in the strongest effects, with inhibition exceeding 70 % even at 4 °C and approaching complete suppression at 25 °C. This concentration–response behavior agrees with previous work on *L. plantarum* supernatants acting against *Listeria monocytogenes* and *Salmonella enterica* serovar Enteritidis (Xi et al., 2025).

Temperature also substantially modulated antibiofilm activity. Inhibition increased with temperature at 2 × MIC and 4 × MIC, whereas at 8 × MIC the impact of temperature was less evident, as high CFS levels dominated the response. Nevertheless, under refrigeration (4 °C) the inhibitory effect was still lower than at 25–37 °C, in line with previous observations that antibiofilm activity of disinfectants and LAB-derived organic acids tends to decrease at low temperatures (Abdallah et al., 2015; Carvalho et al., 2022). Even so, inhibition still exceeded 70 % at 8 × MIC, which is particularly relevant for food biopreservation, given the ability of *R. glutinis* to form biofilms under refrigeration (Gabier et al., 2005; Zhang et al., 2007).

When comparing the two carbon sources, CFS-Ar consistently exhibited numerically greater inhibition than CFS-Gl at lower concentrations (2–4 × MIC), although these differences were not statistically significant. At 8 × MIC, both supernatants achieved near-complete inhibition, indicating that their overall antibiofilm efficacy was comparable across all concentrations. This suggests that while the carbon source may introduce minor variations in potency, both arabinose- and glucose-derived CFSs demonstrate similarly strong preventive antibiofilm effects against *R. glutinis* biofilms.

The strong antibiofilm and antifungal activity observed even at 4 °C highlights the potential of *L. plantarum* S61 supernatants as natural preservatives for refrigerated foods. Their acid tolerance and thermostability activity make them suitable for application in minimally processed or chilled products where chemical preservatives are undesirable. Such properties could also be exploited in antifungal coating formulations or bioprotective cultures for dairy, meat, and plant-based foods.

#### 3.7.2 Curative antibiofilm activity

**Fig. 7:**
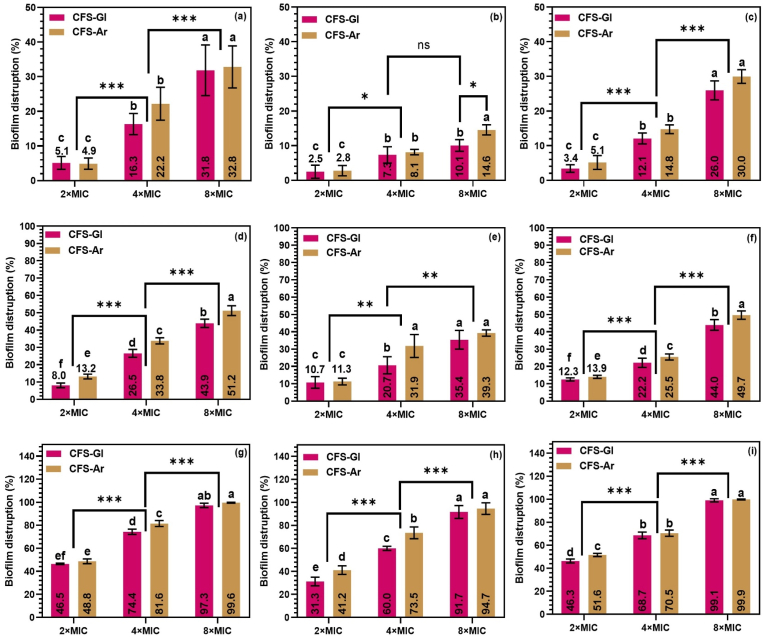
Curative antibiofilm effect of *Lactiplantibacillus plantarum* S61 cell-free supernatants (CFS-Gl and CFS-Ar) against pre-formed *Rhodotorula glutinis* UMP22 biofilms at different incubation temperatures and exposure times. (a–c) 2 h contact at 4 °C, 25 °C, and 37 °C; (d–f) 4 h contact at 4 °C, 25 °C, and 37 °C; (g–i) 24 h contact at 4 °C, 25 °C, and 37 °C. Data are presented as mean ± SD (n = 4)**.** Different lowercase letters (a–f) and the asterisk (∗) indicate statistically significant differences between groups at *p* < 0.05.

The curative antibiofilm activity of *L. plantarum* S61 cell-free supernatants (CFS-Gl and CFS-Ar) against pre-formed *R. glutinis* UMP22 biofilms was evaluated as a function of exposure time, concentration, and incubation temperature (Fig. 7). Exposure time exerted a significant effect on biofilm disruption, with all treatments showing progressively increased removal over time at all temperatures. After 24 h of treatment, the highest concentration (8 × MIC) of both CFS-Gl and CFS-Ar resulted in almost complete biofilm eradication (Fig. 7g–i). This delayed response may be attributed to the protective nature of the extracellular polymeric substances (EPS) matrix, which can limit the rapid diffusion of antifungal metabolites. In addition, weak organic acids such as lactic, acetic, and phenyllactic acids require time to accumulate intracellularly in sufficient quantities to lower cytoplasmic pH and disrupt enzymatic activity (Flemming and Wingender, 2010; Zhang et al., 2021). Prolonged exposure may also induce transcriptional reprogramming, including the downregulation of adhesion- and biofilm-associated genes, as previously observed in *Candida* biofilms treated with *Lactobacillus* supernatants (Poon and Hui, 2023).

The concentration of the supernatants also played a critical role, following a clear dose-dependent trend consistent with the preventive assays. Biofilm removal was limited at 2 × MIC, moderate at 4 × MIC, and most pronounced at 8 × MIC. This pattern agrees with prior reports showing that higher concentrations and longer exposure times enhance biofilm eradication (Wang et al., 2018; West et al., 2018).

Temperature further influenced curative activity, underscoring the importance of environmental optimization for potential applications. Unlike the preventive assays (where higher temperatures enhanced inhibition), the kinetics of biofilm removal reflected the intrinsic biofilm-forming capacity of *R. glutinis* at each temperature. As previously shown in Fig. 5, biofilm development was most pronounced at 25 °C, followed by 37 °C and 4 °C. Consequently, short-term exposures (2 h) produced relatively higher removal at temperatures less favorable to biofilm growth, following the trend 4 °C > 37 °C > 25 °C. After 24 h, however, treatment with 8 × MIC yielded comparable biofilm removal across all temperatures, while lower concentrations continued to exhibit the same temperature-dependent pattern. These results confirm that biofilm removal efficiency depends on the combined effects of time, concentration, and temperature.

Interestingly, although many studies have reported that organic acids exhibit limited antibiofilm activity below room temperature, our results suggest a broader efficacy spectrum. For instance, the antibiofilm effects of organic acids against *Salmonella enterica* serovar Enteritidis, *Escherichia coli*, and *Campylobacter jejuni* were shown to increase with temperature (Carvalho et al., 2022), and enhanced removal efficiency at low temperatures has been achieved using higher disinfectant concentrations or extended contact times (Møretrø et al., 2012). The significant biofilm removal observed at 4 °C in this study indicates that *L. plantarum* metabolites retain antifungal activity under cold conditions, suggesting the involvement of additional compounds beyond organic acids, possibly peptides, biosurfactants, or phenolic derivatives.

Although the antifungal potential of CFS-Ar was generally higher than that of CFS-Gl, no significant differences were observed in curative antibiofilm activity between the two, paralleling the preventive assays. This consistency implies that the principal antifungal metabolites shared by both supernatants are responsible for biofilm disruption in *R. glutinis*.

When compared with conventional antifungal drugs, the activity profile of *L. plantarum* supernatants is distinct. Synthetic antifungals such as fluconazole and amphotericin B act rapidly but are poorly effective against biofilms, requiring high concentrations due to EPS protection and efflux pump activity (Malchikova and Klyasova, 2021; Uppuluri et al., 2011). In contrast, *L. plantarum* supernatants act more gradually but achieve sustained inhibition through multiple mechanisms (organic acids, peptides, and biosurfactants) that collectively lead to extensive biofilm disruption (Zamani et al., 2017). Importantly, LAB-derived metabolites remain active against strains resistant to fluconazole and amphotericin B (Shawky and Hashem, 2021). These findings highlight LAB metabolites as promising, natural, and resistance-free alternatives or adjuncts to conventional antifungal agents for managing fungal biofilms.

## 4 Conclusion

This study indicates that cell-free supernatants (CFSs) produced by *Lactiplantibacillus plantarum* S61 display notable antifungal and antibiofilm activity against *Rhodotorula glutinis* UMP22. The type of carbon source used during fermentation was identified as a major determinant of efficacy, with arabinose-derived supernatants (CFS-Ar) consistently outperforming glucose-based ones (CFS-Gl).

Mechanistic investigations revealed that the inhibitory activity of *L. plantarum* S61 CFSs is multifactorial, combining the effects of heat-stable organic acids and protease-sensitive peptides. Together, these metabolites disrupt fungal membranes, induce intracellular acidification, and disturb metabolic homeostasis. Propidium iodide uptake assays confirmed that membrane permeabilization contributes significantly to the antifungal effect, while the persistence of activity after enzyme treatment and pH adjustments highlighted the involvement of additional metabolites such as phenolic derivatives or biosurfactants.

The combination of CFSs, particularly CFS-Ar, with sub-inhibitory concentrations of cycloheximide produced a marked synergistic inhibition, achieving near-complete growth suppression while substantially reducing the amount of chemical antifungal required. This synergy likely arises from the complementary actions of translational inhibition by cycloheximide and membrane destabilization by LAB metabolites. Such combined strategies hold promise for enhancing antifungal efficacy, minimizing toxicity, and limiting the emergence of resistance.

Antibiofilm assays further demonstrated that both CFS-Gl and CFS-Ar significantly reduced biofilm formation and eradicated established biofilms under a wide range of environmental conditions, including refrigeration (4 °C). This cold-active performance is particularly relevant for food preservation systems, where spoilage yeasts such as *R. glutinis* can persist under low-temperature storage.

Overall, these findings identify *L. plantarum* S61 as a promising source of bioactive metabolites with combined antifungal and antibiofilm activities. Its efficacy under physiological conditions suggests potential not only for food safety applications but also for controlling opportunistic yeasts in clinical contexts. Combining LAB-derived supernatants with reduced doses of conventional antifungals may offer a more sustainable, resistance-mitigating strategy for managing fungal contamination in industrial and biomedical settings. Further studies on chemical characterization, molecular confirmation, and validation in real food matrices are still needed to support practical applications.

## CRediT authorship contribution statement

Sara MOUMNASSI: Formal analysis, Investigation, Methodology, Conceptualization, Writing – original draft. Adem GHARSALLAOUI: Conceptualization, Resources, Writing – review & editing, Supervision. Mohamed BRAHMI: Investigation, Methodology, Writing- Review & Editing. Noure Eddine BENTOUHAM: Writing - Review & Editing. Meryem IDRISSI YAHYAOUI: Writing - Review & Editing. Mohamed TAIBI: Writing - Review & Editing. Bouchra EL GUERROUJ: Writing - Review & Editing. Reda BELLAOUCHI: Writing - Review & Editing. Emilie DUMAS: Conceptualization, Supervision. Abdeslam ASEHRAOU: Conceptualization, Resources, Writing – review & editing, Supervision, Funding acquisition.

## Declaration of competing interest

The authors declare that they have no known competing financial interests or personal relationships that could have appeared to influence the work reported in this paper.
